# Annotations

**Published:** 1889-09-07

**Authors:** 


### Annotations.

**What is the Next Step?**

Only a very few people can have any adequate idea of the toils, anxieties, and disgusts of getting a Bill through Parliament. The Bill may be the most reasonable in the world; it may appeal to justice, common-sense, humanity, and everything else that is good, and yet there will always be some miserable crotchet-monger or obstinate self-assertor who will find difficulties and objections, or make them if they do not exist. In a certain sense a man can have no more thankless work to do than that which is done for the good of the public. If it be true that all men will speak well of a man who does well to himself, it is equally true that many men will speak ill of a man who does well to others. Among the worst and most despicable of human beings are those who, whenever they see a man striving to do a good public work, instantly call out that he has some ulterior personal end to serve. If the present writer were a Dante, he would take care to give such people a very uncomfortable place in a modern Purgatorio. The Rev. Benjamin Waugh has performed the herculean task and endured the innumerable harassments of carrying the Children's Protection Bill through Parliament. The Bill is now an Act, and part of the law of the land. Some few people are ignorant enough to suppose that the Act having become law, all is completed, and nothing remains to be done. The truth is that everything remains to be done. The Act is nothing more than an instrument for doing certain work. The instrument is a good one; considering that it has had to run the gauntlet of two Houses of Parliament, it may be called a very good one. But it can never do any work at all, either good or bad, unless it is put into use, and it is practically certain that it will never be put into use unless Mr. Waugh's society puts it into use. The Society for the Prevention of Cruelty to Children must be regarded as the engine-driver and the stoker of the new Act. But what can either engine-driver or stoker do without coal, and how can coal be obtained without money? In ultimate analysis it is money that will protect the lives and limbs of the helpless little ones. A little money will protect them a little; plenty of money will protect them well. The Hospital has now been long enough in existence for the establishment of a large measure of confidence between writers and readers; and we therefore venture to ask our readers with all possible earnestness to place the claims of this children's society among the very first to which they will respond. We cannot imagine any better use that money can be put to. The offices of the society are at 7, Harper-street, Bloomsbury.

**"The Law of Battle."**

"Almost all male birds," says Darwin, "are extremely pugnacious, using their beaks, wings, and legs for fighting together. . . The smallest of all birds, the humming-bird, is one of the most quarrelsome. With waders, the males of the common water-hen (*Gallinula Chloropus*), when pairing, fight violently for the females. They stand nearly upright in the water and fight with their feet. Two were seen to be thus engaged for half an hour, until one got hold of the head of the other, which would have been killed had not the observer interfered; the female all the time looking on as a quiet spectator." From animals to man is an easy transition. If it is natural for animals to fight, it is natural to man. If the fittest survive in bird conflicts, the fittest will survive in human conflicts. This is natural all the world over, and, therefore, it must be right. Therefore, also, when the weakest go to the wall and the strongest to a dinner with the Lord Mayor, it is all exactly as it should be. Many a man consoles himself for being a selfish scoundrel by such easy philosophy as this. He forgets that bird nature is bird nature, that lion nature is lion nature, and that man nature is man nature. Is there any plainer scientific "law" in creation than this—that all creatures do that which their natural endowments fit them for? The natural endowments of a bird or a lion fit it for following its strongest instincts. The natural endowments of the highest type of man fit him for acting under the control of reason and the impulsion of conscience. The most rigid evolutionists admit this as unquestioningly as the most enthusiastic religionists. The finest products of development are those human creatures in whom reason and conscience are the strongest. The truly scientific man is bound to be truthful, just, regardful of others, rational and moral in the highest degree. He who appeals to science and the law of battle to justify him in selfishness, base indulgence of the passions, injustice and cruelty to his fellow men, appeals to a court which condemns him absolutely and without mercy. "Thou art a man," says that court. "The law of science for thee is the law of man; the law of a perfectly rational and perfectly moral being." In truth, whether a man take science as his teacher, or a reasonable religion, or both, if he have an honest heart and a competent intellect, he is bound to come to the conclusion that man is an animal with intellect and conscience added, and that as each creature is made to obey the highest law of its own being, so is it absolutely incumbent upon man to obey reason and conscience first and before any other law of his nature and life. Vice of all kinds is as hateful to true science as it is to true religion.

**Disease and Will-Making.**

Many questions in law are much complicated by what may be called medical facts and medical opinions. Instead of becoming less complicated they are likely to become much more so, unless either lawyers and the general public are better instructed in medicine, or medical assessors are appointed to act as assistant judges in all cases where medical evidence is to be tendered. Quite recently some very important facts were brought before the Philadelphia Neurological Society, which showed clearly the effect that disease of the kidneys, for example, what is popularly known as "Bright's disease," may have on the mental condition. A man was taken to University Hospital one evening, and when he was seen by the physician he was half asleep and perfectly quiet. He was roused up, and then gave as intelligent an account of himself as the most rational person could have done. All his symptoms pointed to chronic Bright's disease; and there was nothing whatever to indicate that his mental condition was in any way peculiar. Yet, that very night, in the absence of his attendant, he got up, wandered about the ward, and finally jumped out of the window. In a case similar to this the question arose whether or not a certain patient was in a condition to make a will. The only disease that could be found was Bright's disease. But the man's mental state was peculiar and irrational. He believed that his wife and others had designs upon his life, that people were persecuting him; and he could hardly be persuaded to take food. Under treatment the kidneys cleared up, health was restored, and all the mental aberrations disappeared. Other cases of a similar kind are recorded. Now, it is perfectly clear that in all these instances wills made at the acute stage might have been exceedingly unjust, and might, indeed, have expressed the very opposite of what the patient's intentions would have been when quite well. But it is equally certain that if such a case were taken into court the conflict of medical opinion would be startling. How could any ordinary judge, trained only in the law, thread his way rightly through any such case? He could not do it at all. Yet a medical judge, empowered to ask medical questions, would find out at once the nature and severity of the kidney affection, and the kind and extent of the influence it was likely to have upon the brain. What would be all mystery and darkness and professional wrangling to the merely legal jurist, would be clear as noonday to the medical. But is it not the one object of the law to do what is right and just? What really practical objection can be made against medical assessors in medical cases? None, except such as mere prejudice would contend for. The very same reason that causes the State to insist upon a State-paid post-mortem examination in any case of doubtful death ought to induce the State to demand a judicial medical opinion on all medical testimony. If the policeman, who is a State functionary, is compelled to seek medical aid in order to assign the cause of death, so also should the judge be compelled to seek medical aid in order to assess the value of medical evidence. The right judging of medical evidence is as impossible to a lawyer as the discovery of the cause of death is to the policeman; because neither of them has any understanding of the facts with which they have to deal.

